# Orthopaedic Trauma Management in Developing Countries: A Narrative Review of Clinical Practices, Socioeconomic Challenges, and Healthcare Outcomes

**DOI:** 10.7759/cureus.108471

**Published:** 2026-05-08

**Authors:** Arvind Ambedkar, Ravi Diwakar, Agam Kant, Chetan Singh Dhosariya, Gowtham N., Mithul V Mammen

**Affiliations:** 1 Department of Orthopaedics, District Hospital, Shahdol, IND; 2 Department of Orthopaedics, Virendra Kumar Sakhlecha (VKS) Government Medical College, Neemuch, IND; 3 Department of Orthopaedics, Madhya Pradesh Medical Science University, Jabalpur, IND; 4 Department of Orthopaedics, Apollo Sage Hospitals, Bhopal, IND; 5 Department of Pharmacology, Sree Balaji Medical College and Hospital, Bharath Institute of Higher Education and Research, Chennai, IND; 6 Department of Pharmacy Practice, Teerthanker Mahaveer College of Pharmacy, Teerthanker Mahaveer University, Moradabad, IND

**Keywords:** developing countries, epidemiology, health systems, rehabilitation, surgical management

## Abstract

Orthopaedic trauma imposes a disproportionate clinical and socioeconomic burden on developing countries, where rising injury rates intersect with fragile health systems and limited financial protection. This review synthesises evidence from 2015 to 2025 to examine epidemiological patterns, clinical practices, system-level constraints, and socioeconomic determinants shaping orthopaedic trauma outcomes in low- and middle-income countries (LMICs). Using a narrative-synthesis methodology and drawing from major global databases and grey literature, the review identifies persistent deficiencies across the trauma care continuum, including inadequate pre-hospital response, limited diagnostic capabilities, inconsistent surgical management, shortages of trained orthopaedic and rehabilitation personnel, and severely underdeveloped postoperative physiotherapy services. High rates of infection, malunion, non-union, and delayed functional recovery were found to primarily reflect systemic weaknesses rather than intrinsic clinical complexity. The unique contribution of this review lies in its integrated analysis of clinical, infrastructural, and social dimensions-an approach rarely applied collectively in LMIC trauma literature, thereby demonstrating how structural inequities and policy gaps drive preventable disability. The findings underscore the need for context-adapted treatment protocols, strengthened trauma workforce development, national trauma registries, expanded financial protection mechanisms, and coordinated multisectoral policy reforms. By providing a narrative and actionable framework, this review offers critical guidance for policymakers, clinicians, and global health stakeholders working to improve trauma systems and advance equitable, high-quality orthopaedic care in resource-limited settings.

## Introduction and background

Orthopaedic trauma is a major global health issue, with its burden most pronounced in developing nations where injuries frequently result in long-term disability, reduced productivity, and significant socioeconomic consequences [1]. Increasing rates of high-energy trauma, driven by rapid urbanisation, rising motorisation, and poor enforcement of road safety regulations, have led to a growing and largely unmet demand for specialised musculoskeletal care in low- and middle-income countries (LMICs) [2]. In addition to road traffic injuries, occupational accidents, falls, interpersonal violence, and natural disasters contribute to a high incidence of complex fractures [3]. In contrast to high-income settings with well-developed trauma systems and advanced diagnostic and surgical capabilities, LMICs often manage these injuries within resource-constrained systems characterised by limited infrastructure and inequitable access to timely and appropriate care [4].

These constraints have far-reaching consequences [5]. Trauma is a leading cause of mortality and disability among young adults, particularly in the economically productive 18-45 age group in LMICs [6]. Orthopaedic trauma contributes substantially to years lived with disability, often resulting in long-term immobility, deformity, chronic pain, and reduced quality of life [7]. Many of these outcomes arise not only from injury severity but also from avoidable delays in treatment, limited availability of skilled personnel, inconsistent clinical guidelines, and restricted access to surgical implants and postoperative care [8]. The burden extends beyond individuals to families and communities, often resulting in catastrophic health expenditure and cycles of poverty when income-earning capacity is lost [9,10].

**Figure 1:**
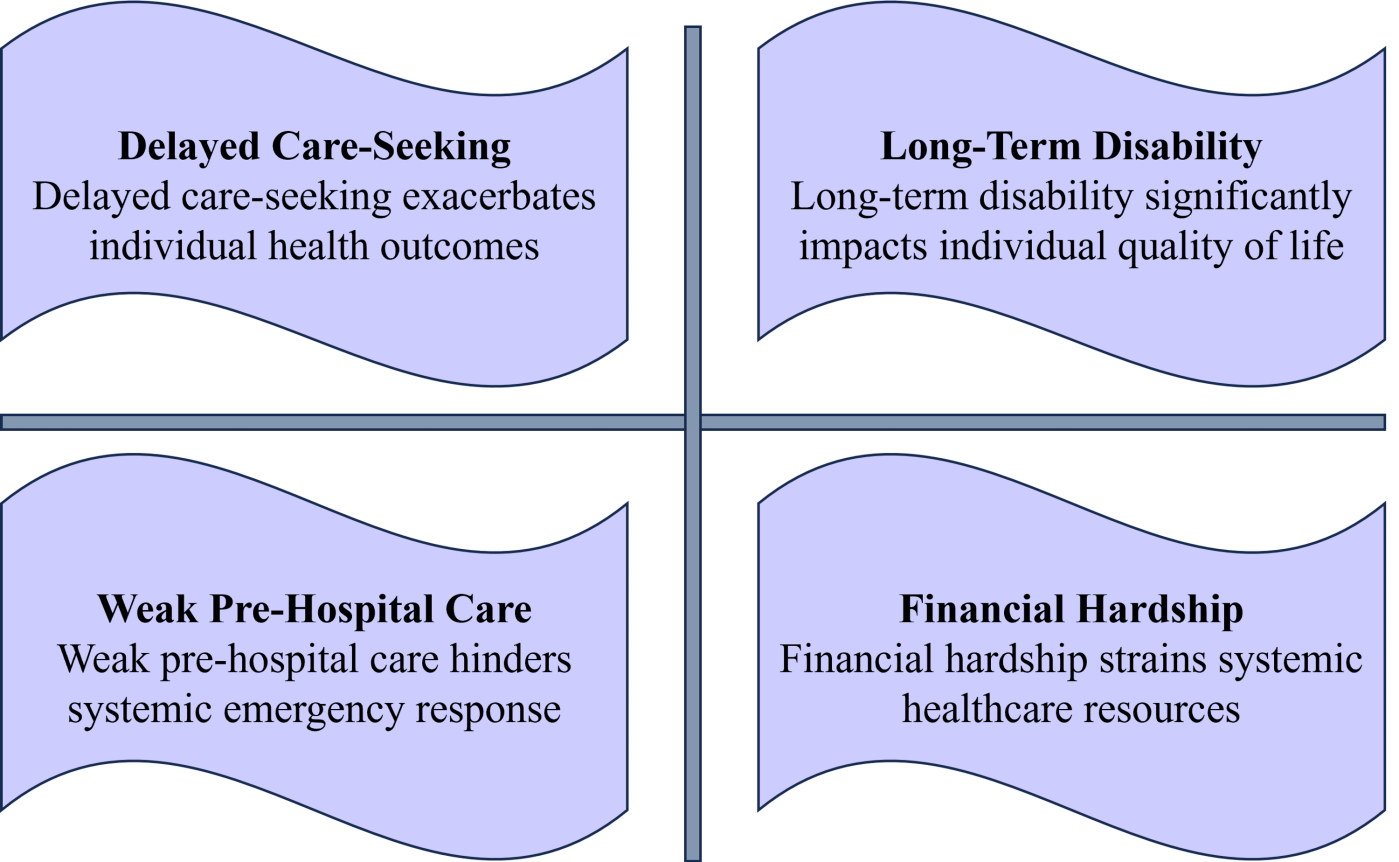
Key factors for poor orthopaedic trauma outcomes in developing countries This image was created by the authors using Microsoft PowerPoint (Microsoft Corp., Redmond, WA).

These findings highlight that orthopaedic trauma care in developing countries is shaped by broader structural and socioeconomic constraints [11]. Pre-hospital care systems are frequently underdeveloped, leading to delays in stabilisation and transport [12]. Emergency services are often limited, with inadequately trained personnel and insufficient ambulance availability [13]. Even after reaching healthcare facilities, shortages of trained orthopaedic surgeons, anaesthesiologists, operating rooms, sterilisation facilities, and imaging resources contribute to treatment delays and reliance on suboptimal management approaches [14,15]. Consequently, complications such as deep infections, malunion, delayed union, and non-union are more common, resulting in poorer long-term functional outcomes [16]. The key causes of poor orthopaedic trauma outcomes are depicted in Figure 1.

These systemic challenges are multifactorial and interrelated. Delayed care-seeking behaviour, influenced by financial constraints, limited awareness, and reliance on informal or traditional care providers, significantly worsens outcomes [5]. Weak pre-hospital systems and high out-of-pocket expenditure further restrict timely access to definitive care [3]. Limited public awareness regarding injury severity, early intervention, and rehabilitation contributes to delayed presentation and poor adherence to treatment protocols [14]. Workforce distribution remains uneven, with specialised services concentrated in urban centres, leaving rural populations underserved [9]. Socioeconomic and cultural factors further complicate trauma care delivery [17]. Out-of-pocket financing often makes surgical care inaccessible, leading to delayed presentation, treatment discontinuation, or reliance on unproven therapies [18,19]. Geographic barriers, including poor transport and remote living conditions, further delay access and follow-up [20]. Cultural perceptions of surgery, pain, and disability influence treatment acceptance and adherence to rehabilitation [21]. These challenges are compounded by the limited availability of trained physiotherapists and specialised rehabilitation services, restricting functional recovery and reintegration [22].

Financial burdens are further increased by indirect costs such as transportation, loss of income, and long-term caregiving. Although government initiatives such as Ayushman Bharat aim to improve financial access, gaps in awareness and implementation persist, particularly in rural populations [3]. Orthopaedic trauma also affects vulnerable groups, including paediatric and geriatric populations, who require specialised management and rehabilitation strategies that remain underdeveloped in resource-limited settings [5,7]. Despite increasing research, existing evidence remains fragmented and context-specific. A comprehensive synthesis integrating epidemiological patterns, health system limitations, clinical practices, and socioeconomic determinants is required to identify cross-cutting gaps and inform effective interventions. This review aims to synthesise current evidence on orthopaedic trauma management in developing countries through a multidimensional approach, examining how clinical practices, health system preparedness, and socioeconomic factors interact to influence outcomes while identifying strategies to strengthen trauma care systems.

Objectives of the review

This study is aimed to (1) synthesise available evidence on orthopaedic trauma patterns and management practices in developing nations; (2) identify system-level, socioeconomic, and clinical determinants influencing access to care and patient outcomes; (3) examine gaps in clinical protocols, healthcare infrastructure, policy support, and rehabilitation services; (4) highlight effective models and innovations suitable for resource-constrained settings; and (5) provide evidence-based, practical recommendations to improve orthopaedic trauma care in LMICs.

## Review

Methodology

The present review utilises a narrative-synthesis approach to examine orthopaedic trauma management in developing countries, based on literature published between 2015 and 2025. This timeframe was selected to capture recent developments in trauma epidemiology, surgical practices, and health system responses in LMICs, ensuring relevance to current clinical and policy contexts. Relevant studies were identified through a structured search of major databases, including PubMed, Scopus, Web of Science, Embase, and the Cochrane Library, along with World Health Organisation reports and grey literature such as governmental publications, NGO reports, and trauma registry data. The search strategy incorporated combinations of MeSH terms and keywords related to orthopaedic trauma, LMICs, fracture management, healthcare systems, and socioeconomic determinants.

Studies were included if they addressed clinical or system-level aspects of orthopaedic trauma care and were relevant to LMIC settings. Evidence from higher-income settings was considered where findings were transferable. A range of study designs, including quantitative, qualitative, and review-based evidence, was included to provide a comprehensive perspective. Inclusion criteria comprised studies focusing on orthopaedic trauma management, healthcare system factors, or patient outcomes relevant to LMICs, published in English between 2015 and 2025, and providing clinical, epidemiological, or health system insights. Exclusion criteria included studies conducted exclusively in high-income settings without contextual relevance to LMICs, articles focusing on non-trauma or unrelated biomedical topics, and publications lacking sufficient methodological or contextual detail to inform the review objectives. Findings were synthesised using a qualitative thematic approach to identify key patterns, system-level challenges, and emerging strategies in orthopaedic trauma care.

Epidemiology of orthopaedic trauma in developing countries

Orthopaedic trauma epidemiology in developing nations is a fast-changing reality characterised by demographic trends, unplanned urbanisation, and changing trends of injury mechanisms [23]. It is always evidenced that young adults aged 18-45 years are at a disproportionate risk of trauma, and the productivity of this group is the key to household income and the state of the economy as a whole [9,24]. The age distribution (preponderance of the trauma group) increases the socioeconomic consequences in the long term, as even minor-to-moderate traumas can interfere with work, and poverty can be further extended [12]. Most of the trauma cases are male, which is linked to more occupational risks, risky driving habits, and physically strenuous work industries [7].

**Figure 2:**
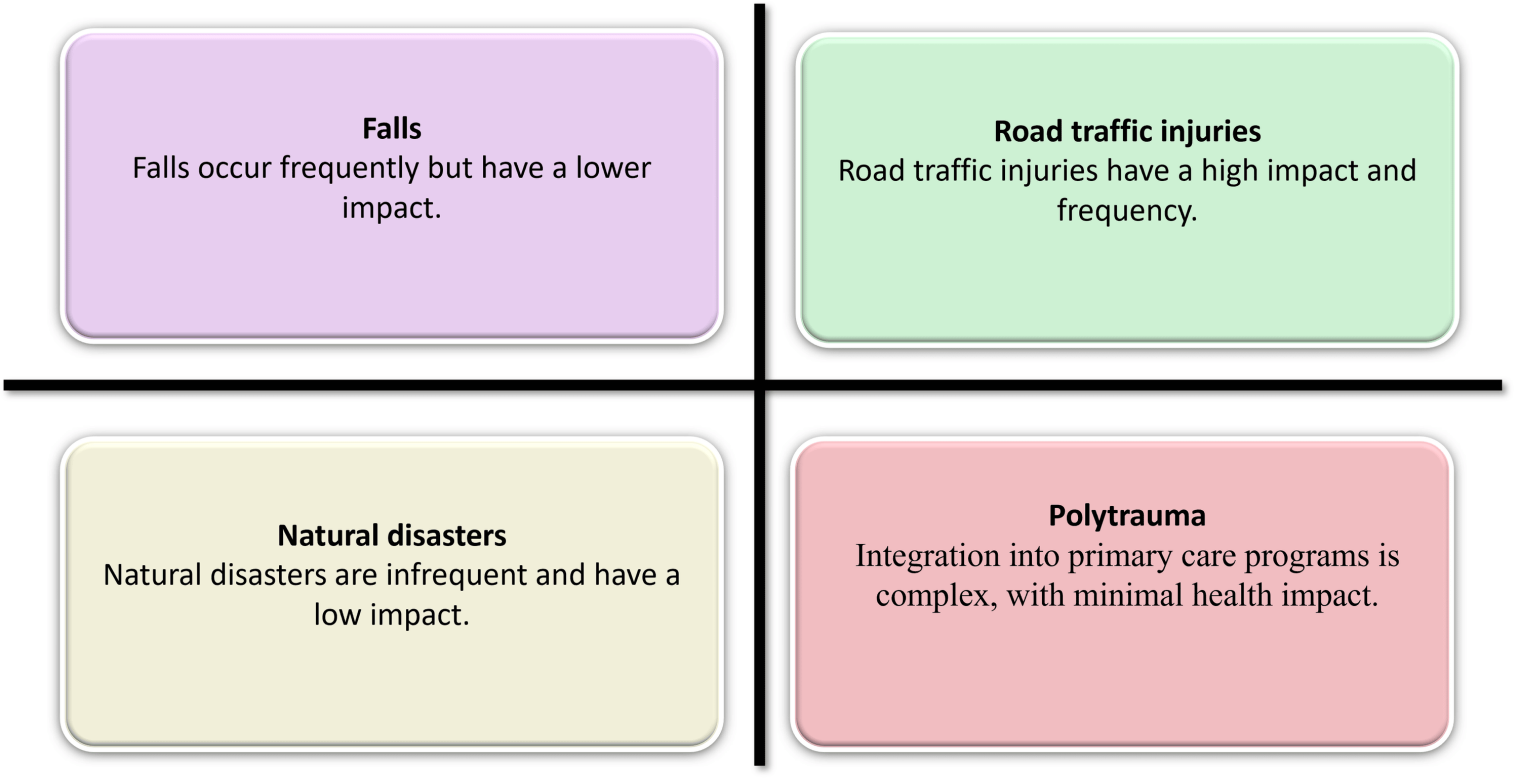
Frequency and impact of major trauma mechanisms This image was created by the authors using Microsoft PowerPoint (Microsoft Corp., Redmond, WA). Source: Refs [1,3,26].

Trauma of the orthopaedic profession is associated with road traffic injuries, which are the most responsible cause of orthopaedic trauma in relation to the use of more motorcycles, overcrowded transport networks, lack of proper implementation of safety laws, and the condition of the roads [25]. Mostly due to these high-energy processes, serious injuries, such as fractures of long bones, disruptions of the pelvis, and open fractures and polytrauma, are often highly severe and need advanced surgical treatment [16]. Other causes, like falls among the elderly and paediatrics, industrial and agricultural accidents, interpersonal violence, and natural disasters, also strain health systems [4]. The open tibial fractures and complicated femoral fractures seem to be the most prominent in LMICs, as they have a delayed presentation and dangerous environmental factors [26]. Comprehensively, the epidemiological data point to an increased trend in preventable injuries and emphasise the immediate necessity of enhanced road safety procedures, workplace precautions, and trauma prevention methods at population levels [27]. Figure 2 demonstrates the patterns of impact of major trauma mechanisms and their frequency.

Healthcare system readiness and infrastructure gaps

Despite the rising burden of trauma, healthcare systems in most developing countries remain inadequately prepared to manage the increasing complexity of orthopaedic injuries [14]. A major gap exists at the earliest stage of the trauma pathway, where pre-hospital care systems are often fragmented or absent [3]. Limited ambulance availability, prolonged response times, and insufficiently trained first responders result in patients being transported without appropriate resuscitation, immobilisation, or wound care, thereby adversely affecting subsequent clinical outcomes [19]. These limitations are further compounded within hospital settings, where infrastructural deficiencies restrict effective trauma care delivery [8]. Many LMICs lack dedicated trauma centres, and general hospitals frequently operate with overcrowded emergency departments, poorly developed triage systems, and inadequate critical care support [11]. Diagnostic imaging services, essential for accurate fracture classification and treatment planning, remain inconsistently available, particularly in rural and semi-urban areas where radiographic facilities may be absent or outdated [6]. Limited access to advanced imaging modalities such as CT and MRI further constrains the management of complex injuries and contributes to variability in clinical decision-making [17].

Surgical infrastructure represents a critical bottleneck in the trauma care continuum [4]. Operating theatres are often limited in number and functionality, with frequent equipment failures, inconsistent power supply, and unreliable availability of sterile instruments and implants [13]. These constraints lead to delays in definitive surgical fixation, increasing the risk of postoperative infections, malunion, and prolonged hospitalisation [1]. Inadequate sterilisation practices further elevate the incidence of surgical-site infections [20]. Additionally, the chronic underdevelopment of rehabilitation services restricts functional recovery, contributing to long-term disability among trauma patients [7]. Taken together, these system-level deficiencies highlight that improvements in isolated components of healthcare infrastructure are insufficient in the absence of coordinated system strengthening. Persistent gaps in pre-hospital services, hospital capacity, surgical resources, and rehabilitation continuity continue to limit effective utilisation of available facilities, thereby sustaining poor trauma outcomes in LMICs [16].

Workforce challenges: shortage of skilled orthopaedic surgeons

Workforce capacity remains one of the most persistent challenges in orthopaedic trauma care in developing nations [28]. Orthopaedic surgeon-to-population ratios in most LMICs are substantially lower than global averages, with the most pronounced shortages observed in rural and remote areas [9]. This imbalance often compels general surgeons or non-specialist clinicians to manage complex trauma cases, leading to variability in clinical practices and increased complication rates due to limited specialised training [15]. Advanced procedures such as internal fixation, fracture arthroplasty, and complex pelvic or acetabular reconstruction are not widely available, as training opportunities are largely concentrated in a few urban academic centres [29]. This geographic concentration of expertise further restricts access to specialised care in underserved regions [6]. In addition, the migration of trained orthopaedic surgeons to high-income countries, driven by better working conditions and career opportunities, continues to exacerbate domestic workforce shortages [12].

The workforce deficit extends beyond surgeons to include anaesthesiologists, physiotherapists, occupational therapists, and orthopaedic clinical officers, all of whom are essential for comprehensive trauma care [30]. High patient volumes in resource-constrained facilities frequently lead to clinician fatigue and burnout, reducing adherence to evidence-based practices and compromising care quality [18]. Although international collaborations, visiting fellowships, and task-sharing initiatives have contributed to capacity building, these efforts remain insufficient to meet the growing demand for trauma services [31]. Sustainable improvement in trauma care delivery requires long-term investment in workforce development, including expansion of training programmes, improved retention strategies, and equitable distribution of skilled professionals across urban and rural settings. Without addressing these systemic workforce limitations, disparities in access to quality trauma care and variability in clinical outcomes are likely to persist [32].

Pre-hospital and emergency care limitations

Pre-hospital and emergency care are essential components of an effective trauma system; however, these elements remain underdeveloped within orthopaedic trauma pathways in developing countries [33]. A critical gap exists at the scene of injury, where structured emergency response systems are often limited or absent [14]. Ambulance networks are frequently inadequate in coverage, poorly coordinated, and constrained by insufficient funding, resulting in delayed response times that worsen initial injuries and reduce survival chances [7]. Consequently, patients are often transported using personal vehicles or informal means that lack basic capabilities such as immobilisation, airway management, and haemorrhage control, increasing the risk of preventable secondary injuries and delayed access to definitive care [12,21].

Workforce limitations further compromise pre-hospital care delivery. Many LMICs lack formally trained paramedics and emergency medical technicians, and first responders often do not receive standardised training in trauma assessment, initial stabilisation, and fracture immobilisation [5,34]. As a result, essential early interventions, including spine protection, bleeding control, and temporary fracture stabilisation, are frequently omitted, contributing to avoidable morbidity [18]. These challenges persist within hospital-based emergency care. Emergency departments are often overcrowded, lack structured triage systems, and operate with insufficient basic supplies such as splints, sterile dressings, and resuscitation equipment [9]. Delays in accessing diagnostic imaging and surgical teams further prolong time to definitive management, leading to poorer clinical outcomes [26]. Collectively, these system-level deficiencies highlight the need for integrated pre-hospital networks, standardised emergency care protocols, and targeted workforce training to reduce preventable morbidity and mortality in trauma patients [35].

Clinical management practices and variability

The clinical management of orthopaedic trauma in developing countries is marked by significant variability in diagnostic and surgical capacity, resource availability, and adherence to evidence-based practices [36]. A key area of inconsistency lies in fixation strategies. External fixation is widely used for the initial stabilisation of open fractures, polytrauma, and long bone injuries; however, in many LMIC settings, it is frequently employed as a definitive treatment rather than as part of a staged surgical approach [11]. This practice is largely driven by limitations in operating theatre availability, implant supply, sterilisation capacity, and surgeon training in advanced internal fixation techniques [22]. As a result, the prolonged use of external fixation is associated with higher rates of infection, joint stiffness, delayed union, and poorer functional outcomes [30]. Access to definitive internal fixation remains uneven across healthcare settings. Plate fixation and intramedullary nailing are increasingly performed in well-equipped tertiary centres, but their availability is constrained by limited implant supply, surgical backlogs, and inadequate infrastructure [37]. Consequently, many patients who would benefit from timely internal fixation are either managed conservatively or experience significant delays in surgical intervention, increasing the risk of malunion and long-term disability [9].

Diagnostic imaging represents another critical source of variability in clinical management. Many district-level facilities lack reliable access to timely radiographic services, while advanced imaging modalities such as computed tomography and fluoroscopy are largely restricted to urban centres [18]. This uneven distribution limits accurate fracture classification, compromises surgical planning, and contributes to inconsistent clinical decision-making and postoperative evaluation [34]. In resource-limited settings, clinicians are often required to modify surgical techniques or improvise materials due to equipment shortages, introducing further variability in the quality of care [38]. In addition, standardised trauma protocols, including ATLS principles and WHO surgical safety guidelines, are not consistently implemented due to training gaps and systemic constraints [15]. These combined limitations result in heterogeneity in clinical practice and contribute to preventable complications and suboptimal outcomes [39,40]. According to Table 1, there are major systemic and resource-based limitations that affect huge variations and suboptimal results of orthopaedic trauma care.

**Table 1:** System-level challenges in orthopaedic trauma care ATLS®: Advanced trauma life support; WHO: World Health Organisation.

Domain	Challenges	Clinical Implications	References
External fixation practices	External fixation is widely used for initial stabilisation but often becomes the definitive treatment because of restricted operating theatre access, inconsistent implant supply, limited sterilisation capacity, and inadequate training in advanced internal fixation.	Increased infection risk, joint stiffness, delayed or non-union, and poorer long-term functional outcomes.	[22]
Internal fixation access	Internal fixation procedures are more frequently available in well-resourced tertiary centres. Access is constrained by uneven implant availability, prolonged surgical wait times, and equipment deficiencies.	Patients experience delays or are treated conservatively, resulting in higher rates of malunion and long-term disability.	[9]
Diagnostic imaging capacity	Many district hospitals lack reliable and timely radiographic services. Advanced imaging, such as computed tomography and fluoroscopy, is limited to selected urban institutions.	Suboptimal fracture classification, compromised surgical planning, inadequate postoperative evaluation, and inconsistent decision-making.	[18]
Technique modification due to resource limitations	Clinicians frequently modify surgical techniques or improvise materials in response to shortages of equipment and consumables.	Variable quality of care and increased risk of intraoperative and postoperative complications.	[38]
Adherence to standardised protocols	ATLS® principles and WHO surgical safety standards are not uniformly implemented due to training gaps, staffing limitations, and systemic barriers.	Reduced standardisation of care processes and a higher likelihood of preventable adverse events.	[15]
Health system constraints	Limited surgical workforce, weak clinical governance, and inconsistent supply chains for implants and consumables restrict service delivery and quality assurance.	Persistent disparities in access, delays in care, and variable treatment outcomes highlight the need for capacity strengthening and context-appropriate trauma management protocols.	[40]

Barriers to post-trauma rehabilitation

The socioeconomic factors have a significant impact on the access of patients to orthopaedic trauma care, and they are also the determinants of the disparity in treatment adherence and long-term patient outcomes [41]. Healthcare financing in most developing nations depends on out-of-pocket payments to a large degree, and the expense of a surgery, implants, diagnostic tests, drugs, and rehabilitation is unaffordable to many members of the legal population [19]. Consequently, patients will often postpone care, refuse recommended surgery, or withdraw treatment too soon [8]. These economic strains are especially important for patients with broken or injured bones, as their recovery may need several operations, the costs of which may become excessive over time [27]. There is also the economic impact on the indirect costs, such as transport, loss of income, and extended caregiving periods, which make homes more vulnerable [36,39].

There are other layers of complexity, which are cultural and sociobehavioural factors [42]. In rural and peri-urban settings, traditional healers can act as the initial call upon first instance; this can be out of long-held community beliefs, distrust of surgical treatments, or misconceptions about biomedical care [14]. These failures to receive timely, proper treatment add up to infection, non-union, and lifelong disability [5]. Geographical inaccessibility is also a significant challenge: most of the communities live miles away from hospitals equipped to deliver trauma care, and inadequate road systems or lack of transportation make it challenging to seek care on time [33]. Follow-ups are not adhered to in most cases after initial treatment due to the high cost of travelling, loss of daily wages, and ignorance of the need for rehabilitation [11].

**Figure 3:**
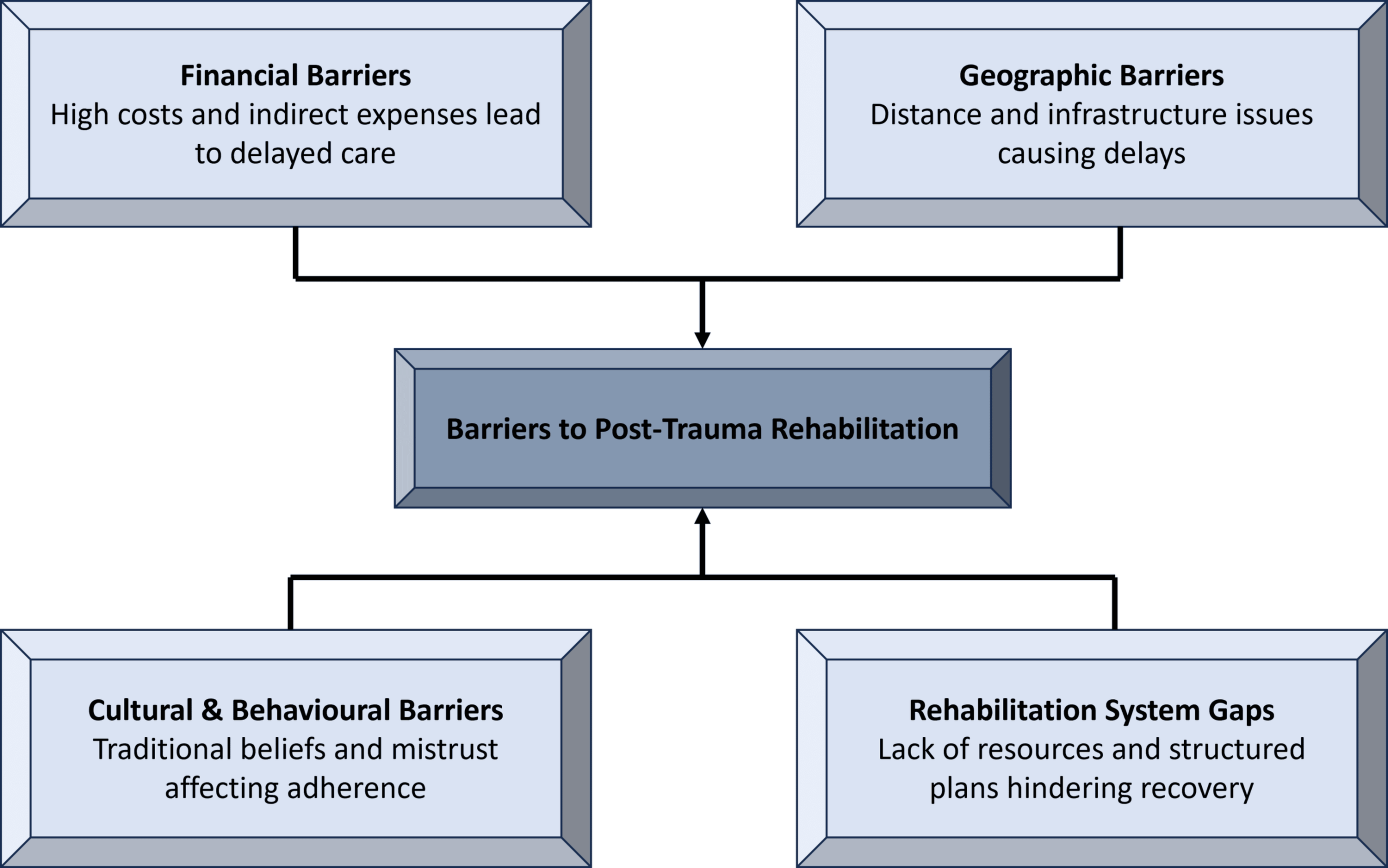
Major barriers to rehabilitation after trauma This image was created by the authors using Microsoft PowerPoint (Microsoft Corp., Redmond, WA).

Many LMICs have limited or no rehabilitation facilities that are required to reinstate functionality [43]. This leads to the release of patients in the homes without organised physiotherapy instructions and hence leads to stiffness, muscle wasting, and poor long-term functioning [22]. These socioeconomic challenges serve to strengthen disparities in trauma effects and the necessity of increased financial protection programmes, culturally sensitive health education programmes, better geographical access to trauma care, and the investment of community-based rehabilitation programmes [44]. Figure 3 demonstrates the key obstacles to effective post-trauma rehabilitation.

Infection burden and complication patterns in trauma care

The incidence and complications of orthopaedic surgery involving trauma care are significantly greater in developing nations than in high-income nations, and this indicates systemic gaps that undermine the quality of care [45]. Overcrowding in hospitals, poor sterilisation procedures, and unreliable access to prophylactic antibiotics, among others, lead to a care environment that predisposes to postoperative as well as hospital-acquired infections [22]. The risks of contamination influence open fractures, which are characterised by their high susceptibility to contamination, especially due to delays in wound debridement, stabilisation, and antibiotic administration, which are frequently caused by surgical backlog, poor staffing, or lack of an available operating theatre [14]. Such limitations lead to increased incidence of deep infections, septic arthritis, and chronic osteomyelitis that contribute to increased hospitalisation and, in most cases, long-term disability or amputation [33].

Other than infections, the high rates of ​​​​​malunion, delayed union, and non-union rates are also caused by delayed intervention and resource constraints [46]. These complications are often a result of poor fracture reduction caused by a lack of access to imaging services, a lack of enough implant options​​​​​, or improvised methods required due to the scarcity of equipment [19]. The socioeconomic strains also enhance the complexity of rehabilitation, as patients are likely to revert to the physically rigorous jobs too soon, thereby compromising the recovery process [7]. Malunion results in deformity, differences in limb length, and biomechanical changes, whereas non-union is usually followed by revision surgery, which is unaffordable to a significant population [27]. All these contribute to a vicious cycle where initial clinical failures lead to the development of chronic complications [41].

Poor compliance with evidence-based guidelines and limited opportunities for continuous professional development further restrict the quality of care provided by clinicians [47]. Uniformity in implementing standardised protocols for fracture management, infection prevention, and postoperative follow-up cannot be promoted, so there is a significant range of clinical outcomes across and within healthcare facilities [11]. Lack of strong quality assurance measures makes this variability worse, and it highlights the importance of having structured protocols, better supply chain management, and a systems-based approach to infection control and complication prevention [32].

Rehabilitation, assistive devices, and long-term functional outcomes

Rehabilitation is an essential part of orthopaedic trauma recovery and is also one of the least developed and least invested aspects of healthcare provision in developing nations [48]. Most hospitals do not have physiotherapy departments or have very limited trained rehabilitation professionals to serve their needs [22]. As a result, rehabilitation services are usually delayed or unavailable [14]. This stagnation causes prolonged immobilisation, joint stiffness, muscle loss, chronic pain, and functional deficits that may continue long after the fracture has healed [36]. Staffing shortages, irregular clinical practices, or insufficient coordination between care teams routinely undermine the quality of early mobilisation, which is a key preventive measure against complications including deep vein thrombosis, pressure ulcers, and contractures [9].

Outpatient rehabilitation is also similarly limited [49]. Physiotherapy services are not provided at a community-based level in many regions, and the financial consequences of regularly commuting to remote hospitals are a huge burden on families [30]. In spite of the existence of services, there is poor adherence to rehabilitation regimens due to a lack of patient education and low awareness of the significance of physiotherapy in areas where services are available [12]. This leads to incomplete functional recovery in many patients, reduced range of motion, and inability to resume employment and other activities as before the injury was sustained [7].

Access to assistive devices for patients who need them is deplorable [18]. There are limited prosthetic and orthotic labs, most of which are located in urban areas and are largely dependent on sporadic external financing [41]. Prosthetic limbs or even tailor-made orthoses are cost-prohibitive for the majority of patients, particularly those who have already exhausted the funds spent on acute care [27]. In addition, there is a lack of follow-up services to maintain or repair the devices, which causes the assistive aids to be underutilised or, more often than not, forgotten over time [33]. These discontinuities negatively affect mobility, independence, and social reintegration [5].

Physical impairment is not the only long-term effect of poor rehabilitation [50]. Survivors of trauma often encounter stigma, work difficulties, and social exclusion, particularly in the workplace, where few policies are inclusive of disabled people [16]. There are no social support structures or vocational rehabilitation programmes, which increases the socioeconomic burden of trauma [11]. An all-inclusive plan to enhance functional outcomes must therefore involve increased rehabilitation facilities, enhanced patient education, community-based services, and improved access to assistive device programmes [32]. As illustrated in Table 2, significant rehabilitation lapses at various levels of the system are a significant impediment to functional recovery and long-term outcomes in orthopaedic trauma patients.

**Table 2:** Multidimensional barriers to rehabilitation after orthopaedic trauma in LMICs DVT: Deep vein thrombosis; LMICs: Low- and middle-income countries.

Rehabilitation Domain	Key Challenges	Underlying Causes	Population Groups Most Affected	Impact on Recovery and Function	References
Availability of rehabilitation services	Limited or absent physiotherapy units, an insufficient number of trained rehabilitation professionals, and late initiation of therapy.	Underfunded health systems, a shortage of specialised staff, and inadequate facility planning.	Rural populations, low-income patients, and trauma cases managed in district hospitals.	Prolonged immobilisation, joint stiffness, muscle atrophy, chronic pain, and long-term functional deficits.	[14]
Early mobilisation and inpatient care	Early mobilisation is inconsistently practised; staffing is inadequate; and interdepartmental coordination is weak.	Staff shortages, lack of standardised protocols, and limited training in postoperative rehabilitation pathways.	Polytrauma patients, elderly individuals, and patients with long bone fractures.	Increased risk of DVT, pressure ulcers, and contractures; delayed restoration of mobility.	[9]
Outpatient and community rehabilitation	Limited outpatient and community physiotherapy access, high transportation costs, and low adherence.	Geographic barriers, lack of community-based rehabilitation models, and poor patient education.	People living in remote areas, low-income families, and informal-sector workers.	Incomplete functional recovery, persistent disability, reduced range of motion, and difficulty returning to work.	[7]
Assistive devices and prosthetic services	Scarce prosthetic/orthotic workshops, high device cost, and limited follow-up or maintenance support.	Urban concentration of services, reliance on external funding, and high production and repair costs.	Amputees, patients with severe limb injuries, and individuals below the poverty line.	Abandonment of devices, restricted mobility, reduced independence, and impaired social reintegration.	[33]
Psychosocial and socioeconomic factors	Stigma, weak disability-inclusive policies, limited vocational rehabilitation, and poor social support.	Cultural attitudes, lack of social welfare programmes, and economic instability.	Patients with permanent disabilities, low socioeconomic groups, and socially marginalised communities.	Social isolation, unemployment, long-term financial hardship, and reduced community participation.	[11]
System-level gaps	Insufficient rehabilitation infrastructure, weak community services, inadequate patient education, and poor access to assistive technologies.	Fragmented health systems, limited funding, and the absence of national rehabilitation strategies.	All trauma patients, particularly those in resource-poor regions.	Persistent disability, prolonged recovery timelines, lower independence, and increased socioeconomic burden.	[32]

Deficits in trauma data systems

One of the root causes of the deficiency in enhancing orthopaedic trauma care in developing countries is the absence of reliable and comprehensive data [28]. Trauma registries, which are needed to determine epidemiological patterns, measure outcomes, and inform health system planning, are either small, fragmented, or do not exist in most parts of the Global South [7]. In the absence of standardised and systematically compiled data, policymakers and clinicians have no clear vision of injury patterns, patient demographics, comorbidity rates, or the efficacy of interventions [42]. The lack of national trauma databases also reduces opportunities to assess disparities at regional levels, determine high-risk groups, or develop focused preventive measures [15].

Hospitals have a high rate of inconsistent clinical documentation, which is often paper-based, and this poses a high risk of information loss and discontinuity of care [33]. Follow-up data are also very limited because most patients fail to visit the hospital after the operation due to financial or geographical reasons [19]. This discontinuity makes it difficult to evaluate long-term outcomes, rehabilitation requirements, and patterns of complications, leading to a limited understanding of patient recovery [4].

The capacity to conduct research in LMICs is also minimal owing to insufficient funding, the absence of training in methodology, and limited access to research infrastructure. The available studies are therefore usually small-scale, single-centre, and observational, which hinders their generalisability and effects on the clinical guidelines at the global level [11]. Moreover, barriers such as language and publication costs further restrict contributions from researchers in low-resource settings [24]. Enhancing trauma data systems by means of digital registries, standardised reporting procedures, and capacity building in clinical research is essential to produce robust evidence that can guide policy change and enhance patient outcomes [37].

Trauma policy gaps and governance barriers

Policy frameworks are a determining factor in how trauma systems are formed, but most developing countries either lack an integrated national trauma policy or implement this policy inconsistently [12]. Even where policies exist, they are usually bombarded by chronic underinvestment, poor governance systems, and competing public health priorities [39]. Critical elements such as ambulance networks, trauma centres, workforce development, and rehabilitation services have not kept pace with the increasing injury burden [5]. This has led to fragmented trauma care, where urban versus rural and the public versus the private sectors are experiencing imbalances [27].

Government measures such as road safety awareness programmes, enforcement of helmet and seatbelt laws, and occupational safety regulations have shown beneficial effects when executed well [44]. However​​​​​, enforcement is not entirely steady, and political commitment varies, restricting its long-term effect [18]. To reinforce national policies, legislative reforms, long-term investment, monitoring, and multisectoral cooperation of transport, labour, and urban planning agencies are necessary [32].

The international partnerships have also contributed significantly to the support of such efforts by providing training programmes, surgical camps, equipment donations, and models for developing trauma systems [21]. Organisations such as the WHO, international NGOs, and academic institutions have played a great role in capacity building and standardising clinical protocols [46]. Nevertheless, such efforts generally require external financing and cannot be feasible long term ​​​​​​​unless ingrained in national structures [50]. A successful system fortification will require nations to take leadership positions, formulate context-sensitive policies, and build partnerships with an emphasis on local ownership, long-term capacity building, and integration into national health programmes [35]. As reflected in Table 3, policy failures and poor governance play a critical role in the underdevelopment, lack of coordination, and limited sustainability of trauma care systems in developing nations.

**Table 3:** Policy and governance challenges affecting the trauma system

Policy Domain	Key Challenges	Underlying Causes	Consequences for Trauma Care	References
National trauma policies and governance	Lack of integrated national trauma policies or inconsistent enforcement of existing frameworks.	Weak governance, underfunding, and competing public health priorities.	Fragmented trauma systems, uneven quality of care, and widening urban–rural and public–private disparities.	[27]
Investment in trauma system components	Insufficient investment in ambulance networks, trauma centres, workforce development, and rehabilitation infrastructure.	Fiscal constraints, low prioritisation of trauma systems, and limited long-term planning.	Delays in emergency response, inadequate facility readiness, limited specialist availability, and poor rehabilitation coverage.	[5]
Injury prevention and safety enforcement	Inconsistent implementation of road safety measures, helmet/seatbelt laws, and occupational safety regulations.	Variable political commitment, inadequate law enforcement, and limited public awareness campaigns.	Preventable injuries remain high, the reduced impact of safety policies, and the continued burden on trauma systems.	[18]
Policy strengthening and multisectoral coordination	Limited legislative reform, weak monitoring mechanisms, and insufficient collaboration with transport, labour, and urban planning sectors.	Siloed government structures, lack of cross-sector policy integration, and inadequate regulatory frameworks.	Poor alignment of injury prevention strategies, reduced policy efficiency, and weak national safety governance.	[32]
International partnerships and external support	Dependence on donor-funded training, surgical camps, equipment donations, and externally developed trauma system models.	Reliance on short-term funding, limited local ownership, and a lack of integration into national strategies.	Unsustainable programmes, inconsistent capacity building, and limited long-term system strengthening.	[46]
Local leadership and sustainability	Insufficient national leadership in developing context-specific trauma policies and integrating external initiatives.	Limited strategic planning capacity, absence of national stewardship, and inadequate institutional frameworks.	Reduced policy sustainability, poor continuity after donor withdrawal, and limited long-term progress in trauma system development.	[35]

Limitations and future directions

The literature on the management of orthopaedic trauma in developing nations is limited by inconsistencies in methodologies and structural limitations that hamper the reliability and usefulness of the existing literature. A large proportion of the literature is based on small, single-centre, retrospective studies with heterogeneous designs, making it hard to make generalisable conclusions and set standardised benchmarks. Under-reporting of injury patterns and complications, along with hindering documentation practices, disjointed data systems, and the lack of national trauma registries, further contribute to this gap. Moreover, socioeconomic and cultural determinants of care, which are decisive variables influencing patient pathways, are understudied, and the absence of long-term follow-up does not provide any information on the functional outcomes, reintegration, and chronic disability. Collectively, all of these gaps hamper the creation of holistic, context-specific trauma care models for low-resource environments.

The way ahead requires a comprehensive strategy that enhances both evidence generation and system performance. Standardisation of trauma protocols based on resource limitations, universal access to low-cost implants, and enhancement of perioperative infection control are critical to the quality of clinical services. Timely and effective care can be greatly promoted through investments in pre-hospital services, coordinated referral networks, and continuous professional training. Equal importance should be given to policy interventions such as expanding insurance coverage, special injury prevention interventions, and long-term collaboration between the government and the private sector to curtail socioeconomic inequities. Most importantly, the creation of national trauma registries and the encouragement of multicentre, LMIC-based research will produce high-quality, contextually informed evidence that can be used to improve trauma management in the long term.

## Conclusions

The issue of orthopaedic trauma in developing nations remains a burning population health issue due to increasing injury predilections, a weak health care system, and existing socioeconomic disparities. This review provides a distinctive literature contribution by providing an integrated, cross-sectoral synthesis related to the connection of epidemiological patterns with system-level limitations, clinical variability, and long-term functional outcomes - areas that are frequently studied separately. It predicts the role of structural vulnerabilities in many avoidable complications in low-resource environments; by consolidating evidence throughout the continuum of trauma care, it was found that many clinical constraints are not the main drivers of such complications. The analysis also identifies important but understudied issues, such as access to rehabilitation, socioeconomic factors, and the lack of a state-of-the-art trauma data system. In the future, it will be necessary to enhance trauma infrastructure, workforce capabilities, and financial protection systems, as well as to create national registries and care pathways suitable to the context. By defining these mutually supporting priorities, this review can help develop a coherent framework to inform policy development, resource distribution, and further studies aimed at achieving equitable and high-quality orthopaedic trauma care in developing countries.

## References

[1] Christie SA, Dickson D, Mbeboh SN (2020). Association of health care use and economic outcomes after injury in Cameroon. JAMA Netw Open.

[2] Laubach M, Hildebrand F, Suresh S (2023). The concept of scaffold-guided bone regeneration for the treatment of long bone defects: current clinical application and future perspective. J Funct Biomater.

[3] O'Hara NN, Slobogean GP, Klazinga NS, Kringos DS (2021). Analysis of patient income in the 5 years following a fracture treated surgically. JAMA Netw Open.

[4] Ang O, Bautista LA, Gerella MC (2025). Understanding the challenges and opportunities for orthopaedic manual physical therapy in Filipino physical therapy practice: an exploratory survey. Philipp J Phys Ther.

[5] Rattan A, Gupta A, Kumar S (2021). Does ATLS training work? 10-year follow-up of ATLS India program. J Am Coll Surg.

[6] Salmond S, Dorsen C (2022). Time to reflect and take action on health disparities and health inequities. Orthop Nurs.

[7] Goddard SD, Jarman MP, Hashmi ZG (2024). Societal burden of trauma and disparities in trauma care. Surg Clin North Am.

[8] Butler EK, Konadu-Yeboah D, Konadu P, Awariyah D, Mock CN (2021). Utility of an orthopaedic trauma registry in Ghana. Ghana Med J.

[9] Benavente-Fernández I, Synnes A, Grunau RE (2019). Association of socioeconomic status and brain injury with neurodevelopmental outcomes of very preterm children. JAMA Netw Open.

[10] Nelson LD, Wilson L, Albrecht JS (2025). Toward more holistic early traumatic brain injury evaluation and care: recommendations from the 2024 National Institute of Neurological Disorders and Stroke traumatic brain injury classification and nomenclature initiative psychosocial and environmental modifiers working group. J Neurotrauma.

[11] McCrum ML, Zakrison TL, Knowlton LM, Bruns B, Kao LS, Joseph KA, Berry C (2024). Taking action to achieve health equity and eliminate healthcare disparities within acute care surgery. Trauma Surg Acute Care Open.

[12] Prerna Prerna, Chadha J, Khullar L, Mudgil U, Harjai K (2024). A comprehensive review on the pharmacological prospects of Terpinen-4-ol: from nature to medicine and beyond. Fitoterapia.

[13] Al-Ajlouni YA, Abouzid M, Tanashat M, Basheer AA, Al Ta'ani O, Bilgin-Badur N, Islam M (2024). Temporal trends in lower extremity amputation in Middle East and North Africa (MENA) region: analysis of the GBD dataset 1990-2019. Int J Equity Health.

[14] Shivasabesan G, Mitra B, O'Reilly GM (2018). Missing data in trauma registries: a systematic review. Injury.

[15] Kiwinda LV, Kocher SD, Bethell MA, Taylor ED, DeBaun MR, Péan CA (2025). Relationship between social determinants of health and patient outcomes after orthopedic trauma. Orthop Clin North Am.

[16] Nagata K, Yamada K, Shinozaki T (2022). Effect of antimicrobial prophylaxis duration on health care-associated infections after clean orthopedic surgery: a cluster randomized trial. JAMA Netw Open.

[17] Bommakanti K, Feldhaus I, Motwani G, Dicker RA, Juillard C (2018). Trauma registry implementation in low- and middle-income countries: challenges and opportunities. J Surg Res.

[18] Chadha J, Ahuja P, Mudgil U, Khullar L, Harjai K (2024). Citral and triclosan synergistically silence quorum sensing and potentiate antivirulence response in Pseudomonas aeruginosa. Arch Microbiol.

[19] Abdelwahab SI, Elhassan Taha MM, Duarte AE, Jan M, Hassan W (2024). The neurosurgical research progress of 98 low and lower middle-income countries from 1928 to 2024. World Neurosurg.

[20] Kushchayev SV, Wiener PC, Teytelboym OM, Arrington JA, Khan M, Preul MC (2019). Percutaneous vertebroplasty: a history of procedure, technology, culture, specialty, and economics. Neuroimaging Clin N Am.

[21] Nzasabimana P, Ignatowicz A, Alayande BT (2023). Barriers to equitable access to quality trauma care in Rwanda: a qualitative study. BMJ Open.

[22] Chin MH, Afsar-Manesh N, Bierman AS (2023). Guiding principles to address the impact of algorithm bias on racial and ethnic disparities in health and health care. JAMA Netw Open.

[23] Izadi Z, Li J, Evans M (2021). Socioeconomic disparities in functional status in a national sample of patients with rheumatoid arthritis. JAMA Netw Open.

[24] Dasa V, Mihalko W, Rivadeneyra A (2024). Innovations in Genicular Outcomes Registry (IGOR): protocol for a real-world registry study of treatments for knee osteoarthritis. Ther Adv Musculoskelet Dis.

[25] Kim J, Cai ZR, Chen ML, Simard JF, Linos E (2023). Assessing biases in medical decisions via clinician and AI chatbot responses to patient vignettes. JAMA Netw Open.

[26] Guest R, Tran Y, Gopinath B, Cameron ID, Craig A (2017). Psychological distress following a motor vehicle crash: evidence from a statewide retrospective study examining settlement times and costs of compensation claims. BMJ Open.

[27] Ahmed Z, Mohamed K, Zeeshan S, Dong X (2020). Artificial intelligence with multi-functional machine learning platform development for better healthcare and precision medicine. Database (Oxford).

[28] Maxwell J, Blashki G (2016). Teaching about climate change in medical education: an opportunity. J Public Health Res.

[29] Roman-Urrestarazu A, van Kessel R (2024). The global burden of disease epidemiology-when big data impute the nonexistent. JAMA Pediatr.

[30] Xames MD, Topcu TG (2024). A systematic literature review of digital twin research for healthcare systems: research trends, gaps, and realisation challenges. IEEE Access.

[31] Hu FB, Willett WC (2018). Current and future landscape of nutritional epidemiologic research. JAMA.

[32] Park M, Magni N, O'Brien DW (2025). Does osteoarthritis physiotherapy research in South Korea align with the National Institute for Health and Care Excellence guidelines: a systematic review of English and Korean literature. BMC Rheumatol.

[33] Lundberg J, Cars T, Lööv SÅ (2023). Association of treatment-resistant depression with patient outcomes and health care resource utilization in a population-wide study. JAMA Psychiatry.

[34] Magesh S, John D, Li WT (2021). Disparities in COVID-19 outcomes by race, ethnicity, and socioeconomic status: a systematic-review and meta-analysis. JAMA Netw Open.

[35] O'Hara NN, Mullins CD, Slobogean GP, Harris AD, Kringos DS, Klazinga NS (2021). Association of postoperative infections after fractures with long-term income among adults. JAMA Netw Open.

[36] Prodinger B, Taylor P (2018). Improving quality of care through patient-reported outcome measures (PROMs): expert interviews using the NHS PROMs Programme and the Swedish quality registers for knee and hip arthroplasty as examples. BMC Health Serv Res.

[37] Ramkumar N, Colla CH, Wang Q, O'Malley AJ, Wong SL, Brooks GA (2022). Association of rurality, race and ethnicity, and socioeconomic status with the surgical management of colon cancer and postoperative outcomes among medicare beneficiaries. JAMA Netw Open.

[38] Tewari P, Sweeney BF Jr, Lemos JL (2022). Evaluation of systemwide improvement programs to optimize time to surgery for patients with hip fractures: a systematic review. JAMA Netw Open.

[39] Twersky SE, Jefferson R, Garcia-Ortiz L, Williams E, Pina C (2024). The impact of limited English proficiency on healthcare access and outcomes in the U.S.: a scoping review. Healthcare (Basel).

[40] Takáč P (2025). Sports injury rehabilitation: a narrative review of emerging technologies and biopsychosocial approaches. Appl Sci.

[41] Garthus-Niegel S, Radoš SN, Horsch A (2022). Perinatal depression and beyond-implications for research design and clinical management. JAMA Netw Open.

[42] Chen N, Fong DY, Wong JY (2023). Health and economic outcomes associated with musculoskeletal disorders attributable to high body mass index in 192 countries and territories in 2019. JAMA Netw Open.

[43] Caffini G, Battista S, Raschi A, Testa M (2022). Physiotherapists' knowledge of and adherence to evidence-based practice guidelines and recommendations for ankle sprains management: a cross-sectional study. BMC Musculoskelet Disord.

[44] Ashruf OS, Ashruf Z, Luyckx V, Kaelber DC, Sethi SK, Raina R (2024). Sociodemographic disparities in 1-year outcomes of children with community-acquired acute kidney injury. JAMA Netw Open.

[45] Car J, Ong QC, Erlikh Fox T (2025). The digital health competencies in medical education framework: an international consensus statement based on a Delphi study. JAMA Netw Open.

[46] Bloom JE, Andrew E, Dawson LP (2022). Incidence and outcomes of nontraumatic shock in adults using emergency medical services in Victoria, Australia. JAMA Netw Open.

[47] Dobbs TE, Carson AP (2022). The hidden factors associated with poor health outcomes. JAMA Netw Open.

[48] Lau PL, Nandy M, Chakraborty S (2023). Accelerating UN sustainable development goals with AI-driven technologies: a systematic literature review of women’s healthcare. Healthcare (Basel).

[49] Kaur S, Rattan A, Kumar H, Rao S, Kant R, Misra MC (2021). Advanced trauma care for nurses (ATCN): a single-center analysis of trauma nurses knowledge gaps. J Trauma Nurs.

[50] Gilstrap LG, Chernew ME, Nguyen CA (2019). Association between clinical practice group adherence to quality measures and adverse outcomes among adult patients with diabetes. JAMA Netw Open.

